# Monobenzyltin Complex C1 Induces Apoptosis in MCF-7 Breast Cancer Cells through the Intrinsic Signaling Pathway and through the Targeting of MCF-7-Derived Breast Cancer Stem Cells via the Wnt/β-Catenin Signaling Pathway

**DOI:** 10.1371/journal.pone.0160836

**Published:** 2016-08-16

**Authors:** Somayeh Fani, Firouzeh Dehghan, Hamed Karimian, Kong Mun Lo, Siyamak Ebrahimi Nigjeh, Yeap Swee Keong, Rahman Soori, Kit May Chow, Behnam Kamalidehghan, Hapipah Mohd Ali, Najihah Mohd Hashim

**Affiliations:** 1 Department of Pharmacy, Faculty of Medicine, University of Malaya, 50603 Kuala Lumpur, Malaysia; 2 Department of Exercise Physiology, Faculty of Physical Education and Sport Sciences, University of Tehran, 14174 Tehran, Iran; 3 Department of Exercise Science, Sports Center, University of Malaya, 50603 Kuala Lumpur, Malaysia; 4 Department of Chemistry, Faculty of Science, University of Malaya, 50603 Kuala Lumpur, Malaysia; 5 Institute of Bioscience, University Putra Malaysia, 43400 Serdang, Malaysia; 6 Medical Genetics Department, National Institute of Genetic Engineering and Biotechnology (NIGEB), Tehran-Karaj Highway, Tehran, Iran; 7 Center for Natural Products and Drug Discovery (CENAR), Department of Chemistry, Faculty of Science, University of Malaya, Kuala Lumpur, Malaysia; University of Nebraska Medical Center, UNITED STATES

## Abstract

Monobenzyltin Schiff base complex, [*N*-(3,5-dichloro-2-oxidobenzylidene)-4-chlorobenzyhydrazidato](o-methylbenzyl)aquatin(IV) chloride, C1, is an organotin non-platinum metal-based agent. The present study was conducted to investigate its effects on MCF-7 cells with respect to the induction of apoptosis and its inhibitory effect against MCF-7 breast cancer stem cells. As determined in a previous study, compound C1 revealed strong antiproliferative activity on MCF-7 cells with an IC_50_ value of 2.5 μg/mL. Annexin V/propidium iodide staining coupled with flow cytometry indicated the induction of apoptosis in treated cells. Compound C1 induced apoptosis in MCF-7 cells and was mediated through the intrinsic pathway with a reduction in mitochondrial membrane potential and mitochondrial cytochrome *c* release to cytosol. Complex C1 activated caspase 9 as a result of cytochrome *c* release. Subsequently, western blot and real time PCR revealed a significant increase in Bax and Bad expression and a significant decrease in the expression levels of Bcl2 and HSP70. Furthermore, a flow cytometric analysis showed that treatment with compound C1 caused a significant arrest of MCF-7 cells in G0/G1 phase. The inhibitory analysis of compound C1 against derived MCF-7 stem cells showed a significant reduction in the aldehyde dehydrogenase-positive cell population and a significant reduction in the population of MCF-7 cancer stem cells in primary, secondary, and tertiary mammospheres. Moreover, treatment with C1 down-regulated the Wnt/β-catenin self-renewal pathway. These findings indicate that complex C1 is a suppressive agent of MCF-7 cells that functions through the induction of apoptosis, cell cycle arrest, and the targeting of MCF-7-derived cancer stem cells. This work may lead to a better treatment strategy for the reduction of breast cancer recurrence.

## Introduction

Breast cancer is the second most common cancer type that affects women. After lung cancer, it is responsible for the greatest number of cancer deaths among women [1].

Chemotherapy, along with a panel of breast cancer drugs, is the most common treatment for this disease. These drugs are categorized as alkylating agents, cytotoxic antibiotics, mitotic and topoisomerase inhibitors, anti-tumor agents and anti-metabolites [2]. Surgery, radiation therapy, hormone therapy, and bone-directed therapy are the other typical treatments for breast carcinoma [3]. Due to the side effects and the development of resistance to chemotropic drugs, the investigation of new anti-cancer agents from various resources must continue. Based on these consequences of cancer treatment, the inclination towards synthetic compounds has been markedly increased [2].

Organotin derivatives, which are non-platinum metal-based agents, are thought to be very promising potential anti-tumor drug candidates [4]. According to studies in recent years, organotin (IV) complexes with Schiff bases create a high level of cytotoxicity for several human cancer cell lines. Complexes of organotin (IV) with Schiff bases are frequently more effective than some metal-based agents such as cisplatin [5–11]. The composition of the ensuing complex, the amount, the characteristics of the organic groups bound to the tin center and the selection of coordinated ligands affect the biochemical activity of the organotin compound [12–17]. Our understanding of breast tumor development and the improvement in the treatment of this disease has considerably contributed to the elucidation of the molecular mechanisms that are involved in breast cancer metastasis and by unraveling the breast cancer stem cells [18]. Apoptosis, a critical programmed cell death process, is an intrinsic hurdle to cell formation and to the development of tumors [19–21]. Thus, an understanding of the proteins involved in the diverse phases of apoptosis offer chances to find new targets for treatment strategies [22].

Al-Hajj et al showed that CD44^+^/CD24^-/low^ cells within a breast tumor, which are cells that express CD44 protein with faint or negative expression of CD24 protein, were able to form new tumors in NOD/SCID mice when a few hundred of these cells were introduced into a mammary fat pad [23]. These distinct populations of cells, which are characterized by uncontrolled self-renewal and irregular differentiation, are known as breast cancer stem cells (BCSCs) [23–29]. BCSCs are considered to be associated with cancer recurrence and treatment resistance, and thus, they must be eliminated in order to eradicate a tumor and block its relapse [30]. The Wnt/β-catenin pathway plays a critical role in the mammary gland in terms of the self-renewal process of BCSCs [31]. In mammals, cytoplasmic β-catenin translocates to the nucleus and combines with the T-cell factor/lymphocyte enhancer binding factor (LEF/TCF), as a result of the deactivation of GSK-3 by Wnt. This event leads to the transcription of a number of cancer-related genes [32–34]. Intracellular β-catenin levels are controlled by a complex composed of axin, casein kinase 1 (CKI)a, and adenomatous polyposis coli (APC). β-catenin interacts with this complex and is then phosphorylated on three defined amino acids (Ser33/Ser37/Thr41) by GSK-3β via the ubiquitin-proteasome pathway [33,35]. It is well recognized that APC is necessary for the degradation of β-catenin. Phosphorylation of APC by GSK-3β increases the binding of APC to β-catenin [33, 36, 37]. Based on this proposition, the targeting of BCSCs and the Wnt signaling pathway is recognized as a potential strategy for breast cancer therapy [23, 31]. In this study, we present the apoptotic response of our novel drug, organotin complex [*N*-(3,5-dichloro-2-oxidobenzylidene)-4-chlorobenzyhydrazidato](o-methylbenzyl)aquatin(IV) chloride, C1, in cells subjected to complex C1 through an evaluation of the following: caspase 3/7, 8, 9 activities, changes in Bcl2, Bax, and Bad expression, cell cycle arrest, cytochrome c release, changes in Mitochondrial Membrane Potential, MMP, nuclear morphology, and phosphatidyl serine (PS) exposure on the cell surface. Furthermore, the *in vitro* efficacy of our Monobenzyltin Schiff base complex C1 against MCF-7 BCSCs and its ability to suppress the Wnt/β-catenin signaling pathway were investigated.

## Methodology

### Synthesis of benzyltin Complex C1

**Fig 1:**
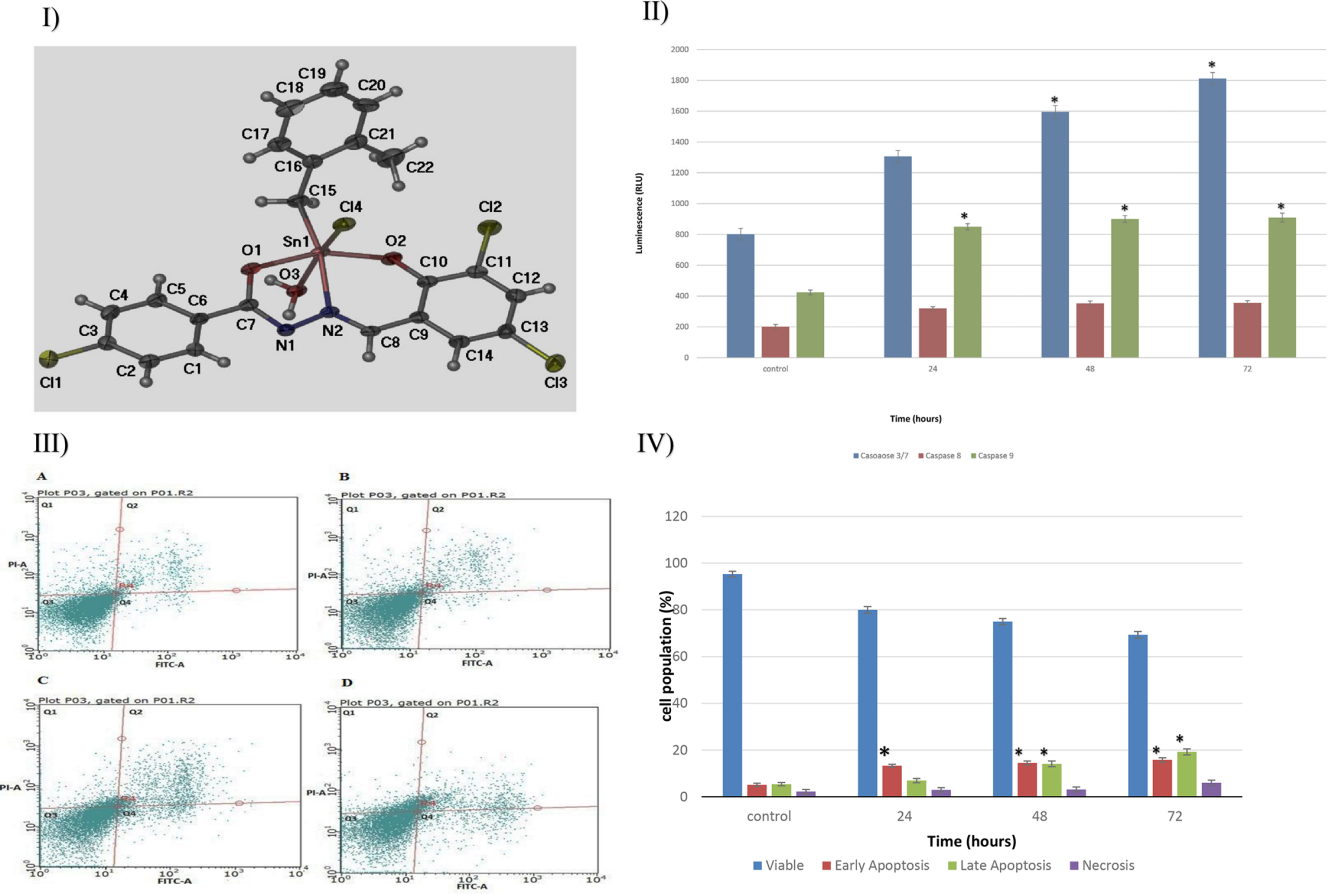
**I) molecular structure of C1, II) caspase activity assay, III) flow cytometric analysis of Annexin V in MCF-7 cells. I**: molecular structure of C1 with numbering scheme. **II:** The luminescence analysis revealed a significant time-dependent expression of caspases 7, 8 and 9 in compound C1-treated MCF-7 cells at a concentration of 2.5 μg/mL (IC_50_).**III**: **(A)** Displays untreated MCF-7 cells; **(B)**, **(C)**, and **(D)** show cells treated with compound C1 at a concentration of 2.5 μg/mL for 24, 48, and 72 hours of treatment. **(E)** Representative bar chart. Data are expressed as the mean ± the standard deviation. Each independent experiment included triplicate treatment groups. Statistical significance was equal to *p < 0.05.

The synthesis and characterization of [*N*-(3,5-dichloro-2-oxidobenzylidene)-4-chlorobenzyhydrazidato](*o*-methylbenzyl)-aquatin(IV) chloride, C1 (Fig 1I) is described in a previously published manuscript [38]. This Schiff base compound was supplied by Dr. Lo Kong Mun of the Department of Chemistry, Faculty of Science, University of Malaya, Kuala Lumpur, Malaysia.

### Cell Culture

The human breast adenocarcinoma cell line MCF-7 was obtained from the American Type Cell Collection (ATCC, Manassas, VA, USA). Cells were cultured in 25 cm^2^ or 75 cm^2^ culture flasks that included a mixture of RPMI-1640, 10% fetal bovine serum (FBS), and 1% penicillin streptomycin. Cells were maintained in a humidified atmosphere (5% CO_2_) at 37°C. Negative control for *in vitro* assays was the untreated medium containing 0.1% vehicle DMSO.

### Staining with Annexin V and Propidium-iodide

An Annexin V FITC Apoptosis Detection Kit I (BD Pharmingen™, Californiam, San Jose, USA) was used to evaluate the apoptotic effect of complex C1. As suggested by the MTT assay from a previous study, an IC_50_ concentration of the compound (2.5 μg/mL) was used to treat MCF-7 cells at a density of 1×10^6^ cells per 25 cm^2^ culture flasks for 24, 48 and 72 hours [38]. Once the treatment of MCF-7 cells was complete, they were collected by centrifugation (2000 rpm for 5 minutes). Any accumulated supernatant was removed, and the remaining cells were washed with 100 μl of binding buffer. Next, 5 μl of both PI and annexin V was added to the cells, which were incubated on ice for 15 minutes in the dark. Following this incubation period, 400 μL of binding buffer was added, and the cells were analyzed with a BD FACSCantoTM flow cytometer (BD Bioscience, San Jose, CA, USA). Untreated cells served as the negative control.

### Cell Cycle Assay

Flow cytometry was used to assess the distribution of cells in the cell cycle. This was accomplished via the treatment and harvesting of MCF-7 cells at a concentration of 1×10^6^ per 25 cm^2^ culture flasks by the same methods as those used for the annexin V assay. The pellet was fixed in ethanol (500 μl) and incubated at -20°C for 1 week. After a week, a PBS-EDTA-BSA solution was created by the addition of 2 mg/mL EDTA to PBS, which was then placed in an autoclave. Next, 10 mL of this mixture was used to dissolve 0.1% BSA. The BSA was then filtered into the PBS-EDTA mixture to produce the final PBS-EDTA-BSA solution. After the addition of 1 mL of the PBS-EDTA-BSA solution to the pellet, the pellet was centrifuged for 5 minutes at 2000 rpm. After centrifugation, a wash buffer composed of 1 g BSA, 20 mg of EDTA, 100 mL of PBS, and 100 mg of Sodium Azide, was used to wash the pellet. Before flow cytometry was conducted, a staining buffer that contained 0.3 μg/mL PI, 0.37 mg/mL EDTA, 1 mL PBS, 1 μl/mL Triton-X100, and 50 μg/mL RNase was used to stain the cells. After they were stained, the cells were placed on ice for 30 minutes.

### Multifactor Cytotoxicity Analysis

The multifactor cytotoxicity analysis was conducted using a Cellomics Multiparameter Cytotoxicity 3 Kit. The first step was to grow the cells to a seeding concentration of 1×10^4^ cells/well in a 96-well plate. After treatment periods of 24, 48, 72 hours, 50 μL of life cell staining solution was placed into each well followed by an incubation at 37°C for 30 minutes. Following the incubation period, the cells were fixed in 50 μL of 4% paraformaldehyde and washed in PBS. Next, 50 μL of the primary antibody solution was added to each well, and the cells were incubated for another hour. Then, 50 μl of the secondary antibody solution was added, and the cells were incubated for an additional hour at room temperature. In order to simultaneously analyze six markers of cell health, an ArrayScan HCS system (Cellomics) was used. The six markers of cell health included changes to cell permeability, cell loss, the localization of cytochrome *c*, DNA content, changes in the mitrochondrial membrane potential, and nuclear morphology. The last row in the plate was used for the negative control. According to the protocol, all solutions used in this experiment were prepared in advance.

### Analysis of Caspase Activity

Several luminescence-based assays were used to evaluate the activities of caspase 3/7, 8, and 9. These assays included the Caspase-Glo 3/7 Assay (Promega), the Caspase-Glo 8 Assay, and the Caspase-Glo 9 Assay. Cells at a density of 1x10^4^/well were plated in 96-well Greiner transparent plates. The plates were incubated for 24 hours to allow cell attachment. As suggested by a previous study, after 24 hours, complex C1 at an IC_50_ concentration (2.5 μg/mL) was used to treat the cells for a period of 24, 48, or 72 hours [38]. The last two rows of the 96 well Greiner transparent plates were used for a blank reaction and negative control. Following a period of 24 hours, 100 μl of caspase-Glo^®^ reagent was added to each well. The caspase-Glo® reagent contained caspase-Glo^®^ substrate, caspase-Glo^®^ buffer, and MG-132 inhibitor. After the reagent was added, a plate shaker was used to shake the plate at 300–500 rpm. The plates were then incubated in the dark at room temperature for 30 minutes. After incubation, an operating microplate reader was used to measure the luminescence.

### Analysis of mRNA Expression by Real time-PCR

An RNeasy Plus Mini Kit (Qiagen, Germany) was used to obtain the total RNA from 10^7^ MCF-7 cells per 75 cm2 culture flasks that were treated for 24, 48, and 72 hours as well as from the untreated cells. Then, 260/280 UV absorption ratios (Gen Quant 1300, UK) were used to assess the RNA purity and concentration. To perform a Two-Step real time PCR, a High Capacity RNA to cDNA Two-Step qRT-PCR Kit produced by Applied Biosystems (USA) was used to convert the total RNA to cDNA. A total of 1 μl of the converted cDNA was used for real time PCR amplification. ß-Actin served as the housekeeping gene. The Bax, Bcl2, and ß-Actin genes were amplified using TaqMan primer and probe sets obtained from predesigned assays provided by Applied Biosystems. A StepOnePlus real time PCR machine was used for the real time PCR reactions. The program for the real time PCR reaction was as follows: reverse transcriptase at 48°C for 15 minutes, activation of ampli Taq gold DNA polymerase at 95°C for 10 minutes, denaturation at 95°C for 15 seconds, and annealing at 60°C for 1 minute. The denaturing and annealing steps were conducted over 40 cycles. The master mix, assays, and StepOnePlus real time PCR machine were all purchased from Applied Biosystems, (Carlsbad, California, USA). The relative quantifications of the PCR products were determined by taking the fold change of each target over the average fold change of the ß-Actin gene according to the comparative Ct (2^-ΔΔCt^) method. The use of this calculation to find the quantification of the PCR products meant that the amplification of the target and the reference gene was quantified in the samples, and therefore, the values for the reference gene were obtained before the measurements and were standardized by GenEx software. The RNA fold changes were calculated using Data Assist v3 software from Applied Biosystems (USA).

### Isolation of Candidate Breast Cancer Stem Cells

A catcher tube-based cell sorter and a flow cytometer (FACSCalibur™, BD Biosciences, Franklin Lakes, NJ, USA) were used to isolate candidate MCF-7-derived breast CSCs by sorting the CD44^+^/CD24^-/low^ cell population. Cells at a concentration of 1×10^7^ cells/mL were stained using 20 μL of both CD44 antibody and CD24 antibody in a 5 mL tube. The following were purchased from BD Biosciences: CD24 mouse anti-human monoclonal antibody [clone SN3], the CD44 mouse anti-human monoclonal antibody [clone MEM-85], fluorescein isothiocyanate [FITC] conjugate, mouse immunoglobulin G2b (FITC), mouse immunoglobulin G1 (R-phycoerythrin), and phycoerythrin conjugate. The tubes were maintained in the dark at room temperature for 45 minutes. The CD44^+^/CD24^-/low^ cell population was recognized using CellQuest Pro software and quadrant analysis.

### An Assay for Non-adherent Mammosphere Formation

A concentration of 10,000 cells/mL of culture medium was used to seed the CSCs derived from MCF-7 cells in six-well ultra-low attachment plates (TPP, Fisher Scientific). Cells possess the capacity for growth and sphere formation in the presence of serum-free Dulbecco’s Modified Eagle’s Medium/F12 medium (Lonza) (DMEM/F12) and certain supplements including 1% antibiotic-antimycotic, 1 μg/mL hydrocortisone, 4 μg/mL gentamicin, 5 μg/mL insulin, 20 ng/mL EGF (Gibco), 20 ng/mL b-FGF (Gibco), and B27 (Invitrogen). Every two days, 1 mL of fresh media containing the above-mentioned components was loaded into each well. In order to investigate the effect of complex C1 on mammosphere formation, the primary culture of MCF-7 CSCs was incubated with diverse concentrations of C1, including 0, 1, 2, and 4 μg/mL. The cells derived from compound C1-treated primary mammospheres were subcultured for secondary and tertiary cultures for each corresponding group without the presence of compound C1. The number of mammospheres that formed was compared with the control and recorded after 5 and 7 days. A Nikon Eclipse TE2000-S microscope with MetaMorph software 7.6.0.0 was used to capture the images.

### Aldefluor Enzyme Assay

High activity of aldehyde dehydrogenase (ADH) in cells is an established hallmark of mammary stem/progenitor cells. In order to measure the activity of ADH, an aldefluor enzyme assay was performed based on the manufacturer’s guidelines for the Aldefluor™ (StemCell Technologies, Herndon, VA, USA) kit. MCF-7 CSCs from the cell cultures were incubated at 37°C in an atmosphere of 5% CO_2_ for 45 minutes. A flow cytometer was used to quantify enzyme activity.

### Analaysis of Protein Markers of Apoptosis, Cell Cycle, Caspases, and the Wnt/β-catenin Self-renewal Pathway

A Nacalai Tesque protein extraction kit was used to extract the total protein from 1×10^7^ MCF-7 cells per 75 cm2 culture flasks (treated with the IC_50_ concentration of compound C1 for 24, 48, or 72 hours). SDS-PAGE (10%) was used to separate 40 μg of protein extract. After it was separated, the protein extract was transferred to a polyvinylidenedifluoride (PVDF) membrane (Bio-Rad). Next, the membrane was blocked with 5% nonfat milk in TBS-Tween buffer 7 (0.12 M Tris-base, 1.5 M NaCl, 0.1% Tween20) for 1 hour at room temperature. After nonspecific proteins were blocked, the membrane was exposed to the β-actin primary antibody (Santa Cruz Biotechnology, CA, USA) at a 1:10,000 dilution, as well as to the Bcl2, Bax, and heat shock protein 70 (HSP70) primary antibodies (Santa Cruz Biotechnology, CA, USA) each at a 1:1000 dilution; the membrane was incubated at 4°C for 8 hours. The primary antibodies for P21, P27 (Anti-p21 antibody and Anti-p27 KIP 1 antibody), caspase 9 (Anti-active Caspase 9 antibody), Bad (Anti-Bad antibody) were used at a 1:1000 dilution (Abcam, USA). The primary antibodies that were used to identify Wnt/β-catenin self-renewal pathways are cyclin D1 (1:5,000), β-catenin, and phospho β-catenin S33+S37 (1:500), GSK3β (1:5,000), phospho GSK3β (1:500). In this study, these antibodies were purchased from Abcam (Cambridge, UK). After washing with TBST, the blots were incubated with goat anti-mouse or goat anti-rabbit secondary antibodies (1:1000) conjugated with alkaline phosphatase (i-DNA, Ocean, NJ, USA) for 45 minutes at room temperature. For visualization, the membranes were washed and subjected to BCIP/NBT solution (Santa Cruz Biotechnology) for a period of 3–20 minutes to detect the target protein bands.

### Statistical Analysis

The data are given as the mean ± the standard deviation of three separate experiments. The Prism statistical software package (Graphpad Software, USA) was used to perform a one-way ANOVA analysis as part of the statistical analysis of the results. The statistical significance was expressed as *p < 0.05.

## Results

### Compound C1 Induced PS Externalization

Interruption or malformation of the cell plasma membrane which is a common property of cancer cells causes the cell construction losing and break of their life cycle [39]. Phosphatidylserine (PS) translocation from the inner side of the plasma membrane to the outer side of the plasma membrane is a typical marker of early apoptosis. The results from previous study of the selective cytotoxic effect of compound C1 on MCF-7 cells inspired us to further inspect the early apoptotic events. An Annexin V FITC kit was used to further investigate the apoptotic effect of complex C1 on MCF-7 cells. The binding of annexin V to the acidic phospholipids found in apoptotic cells can be used to detect the translocation of phosphatidylserine (PS) residues from the inner layer of the plasma membrane to the outer cell surface. After the MCF-7 cells were exposed to 2.5 μg/mL of compound for 24, 48, or 72 hours, an annexin V/PI stain was applied. The flow cytometric analysis revealed that apoptosis occurred in a time-dependent manner (Fig 1IIIA–1D). As many as 2.27% of the untreated cells were necrotic, 5.43% were in the late stages of apoptosis, slightly fewer (5.16%) untreated cells were in the early stages of apoptotic induction, and 87.14% of the untreated control cells were viable. Following incubation with compound C1, the early apoptotic population (Annexin V-positive, PI-negative) increased significantly (*p* < 0.05). After 24 hours, the population increased to 13.35%. The population of early apoptotic cells rose even higher to 14.5% after 48 hours, and it reached 15.81% after 72 hours of incubation. The quantity of late apoptotic cells (positive for both Annexin V and PI) demonstrated continuous growth, with a significant increase from 14.1% to 19.4% between the 48 and 72 hour time points (Fig 1IV). After 72 hours of treatment, the percentage of viable cells (negative for both Annexin V and PI) decreased to 69.30%, while the percentage of necrotic cells (Annexin V-negative, PI-positive) rose significantly from 2.27% in the control group to 5.99% after 72 hours of treatment (Fig 1IV).

### Compound C1 Led to the Arrest of MCF-7 Cells at G0/G1 Phase

**Fig 2:**
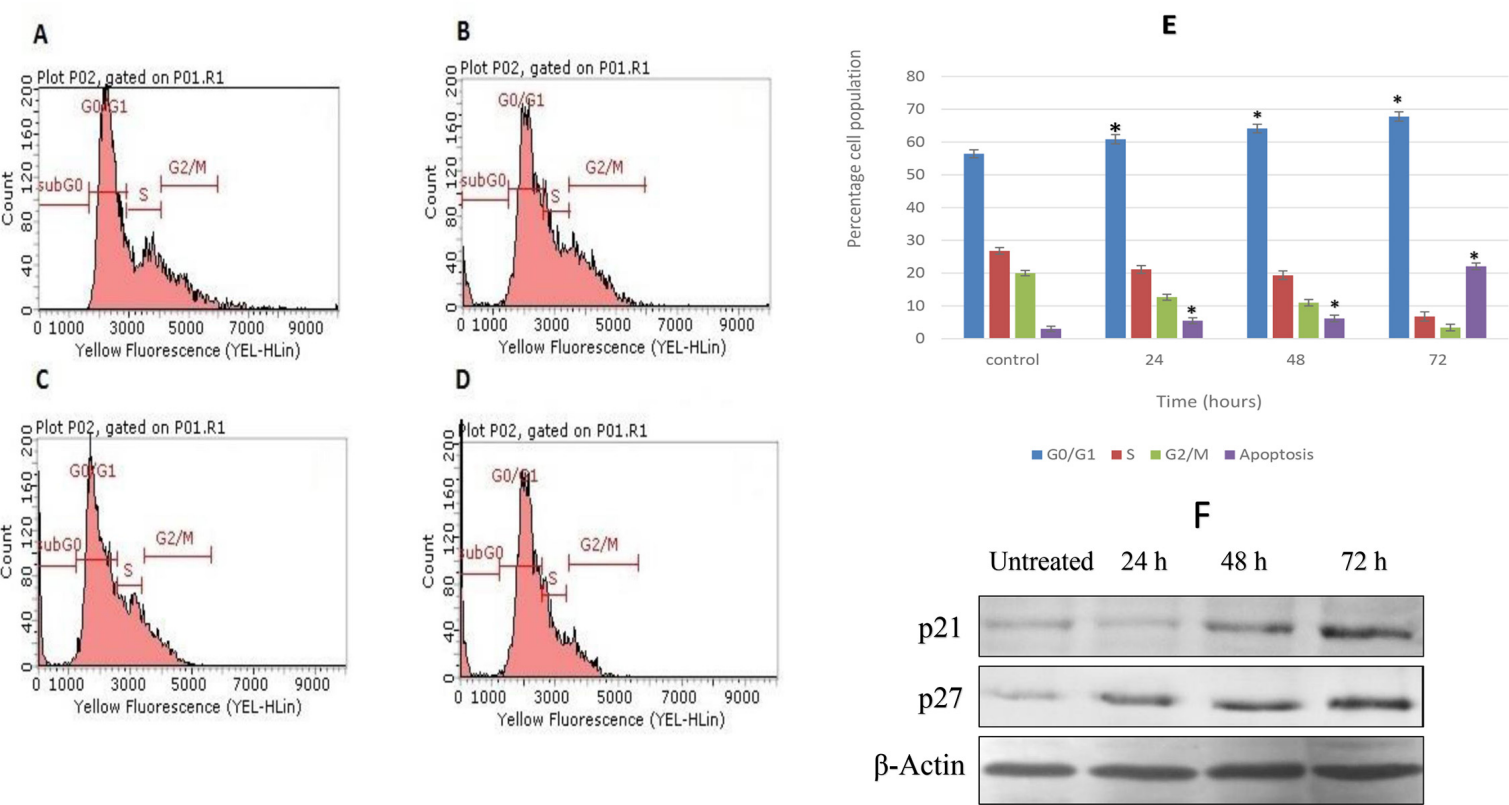
Effect of compound C1 on the cell cycle distribution of MCF-7 cells. (**A**) Shows the untreated MCF-7 cells (control). After treatment with the IC_50_ concentration of complex C1 for (**B**) 24, (**C**) 48 and (**D**) 72 hours, a flow cytometric analysis was performed on treated and untreated MCF-7 cells (**E**). **(F)** Shows the protein expression of the G0/G1 phase specific cyclins p21 and p27. The quantitative analysis indicated cell cycle arrest at the G0/G1 phase. Data are expressed as the mean ± the standard deviation. Each independent experiment included triplicate treatment groups. Statistical significance was equal to *p < 0.05.

Cancer development is often defined with perturbations in the cell cycle process. The improvement in understanding of mechanisms which are involved in oncogenesis and apoptosis, the agents with cell-cycle regulatory effects has gained greater attention to be studied [40]. Following 24, 48, and 72 hours of treatment and staining with PI, a flow cytometric analysis was conducted to determine how compound C1 affected the distribution of cells in the stages of the cell cycle. Fig 2A–2E illustrates the results of this analysis. As shown in Fig 2E, a G0/G1 phase arrest was observed at each time point from 24 to 72 hours in a time-dependent manner. After 24 hours, 60.69% of cells were arrested in G0/G1. After 72 hours, this increased to 67.76%. Concurrently, the percentage of cells in the sub-G0/G1 phase rose significantly from 3.17% (untreated cells) to 5.49% after 24 hours of treatment, after which it increased to 6.2% after 48 hours before rising to 22.08% following 72 hours of treatment (*p* < 0.05). Fig 2E also shows that the cell population in S phase decreased from 26.81% to 6.77%, while the percentage of the population in G2/M phase decreased from 20.01% to 3.41%. The cyclin-dependent kinases (CDKs) have a critical role in cell cycle control through their interaction with cyclins and Cdk inhibitors (CKIs) [39]. The irregular growth of cancer cells is associated with the abnormal activity of CKIs. The members of the CIP/KIP family including proteins p21 (CIP1) and p27 (KIP1) proteins are acknowledged to stimulate cell cycle arrest through the G1 /S CDKs cascade [41]. Since compound C1 caused the G1 cell cycle arrest in treated MCF-7 cells, in the next step the protein expression level of p21 (CIP1) and p27 (KIP1) were assessed. As shown in Fig 2F, compound C1 upregulated the p21 and p27 proteins in a time-dependent method.

### Compound C1 Induced MMP Disruption and Release of Cytochrome *c*

**Fig 3:**
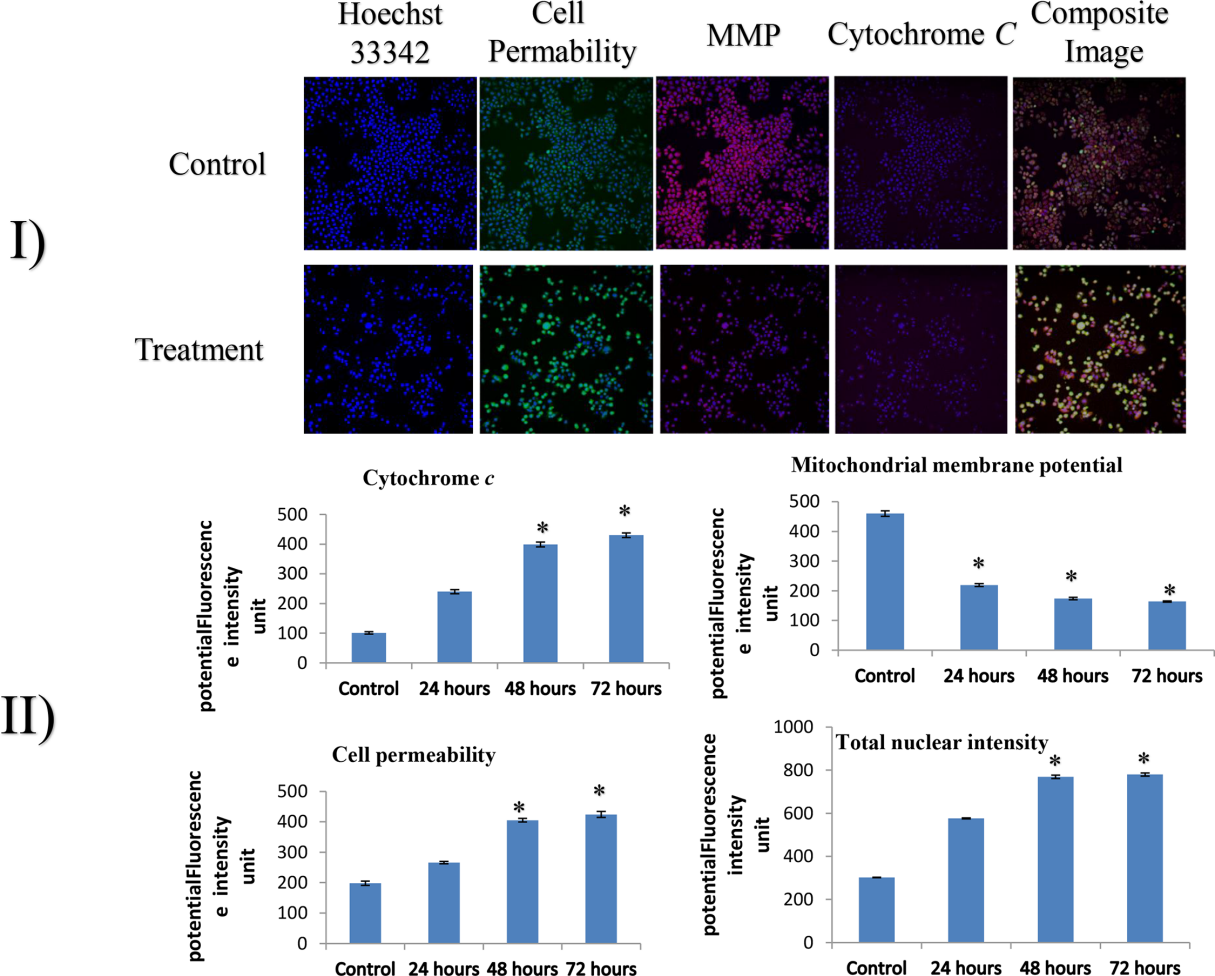
**I) Representative images of MCF-7 cells and II) quantitative analysis of compound C1-mediated apoptosis parameters. I:** Total nuclear intensity, mitochondrial membrane potential, cytochrome *c* release, and cell membrane permeability were detected in MCF-7 cells that were treated with culture medium alone and in cells that were treated with a concentration of 2.5 μg/mL (IC_50_) of compound C1; the cells were stained with Hoechst. The images in each row originate from the same field of the same treatment sample. MCF-7 cells experienced a remarkable reduction in the MMP as well as a significant increase in total nuclear intensity, membrane permeability and cytochrome *c* (magnification 20 x). **II:** Treated MCF-7 cells were examined to measure changes in total nuclear intensity, cell permeability, mitochondrial membrane potential, and cytochrome *c* localization. After they were treated with compound C1 (at the IC_50_), they exhibited a statistically significant loss of mitochondrial membrane potential and a noticeable increase in total nuclear intensity, cell permeability, and cytochrome *c* release from mitochondria. Each experiment was performed in triplicate. The results are expressed as the mean ± the standard deviation. Statistical significance was equal to *p < 0.05.

In order to investigate the possible mechanism of action for inhibition effect of compound C1 on proliferation of MCF-7 cells, we measured the changes in some crucial factors, which are related to apoptosis. Cell loss, cytochrome *c* release, changes in the mitochondrial membrane potential, morphological changes, nuclear size, and changes in cell permeability were measured simultaneously using an ArrayScan HCS system (Cellomics) after 24, 48, and 72 hours of treatment with compound C1 at the IC_50_. The results revealed that changes in the MMP were significantly reduced after 24, 48, and 72 hours of treatment. Cytochrome *c* translocation from the mitochondria into the cytosol during apoptosis also increased significantly in the treated cells for all time periods. Following treatment, a significant increase in total nuclear intensity and cell permeability was clearly observed in MCF-7 cells after 48 and 72 hours of treatment (*p < 0.05) (Fig 3I and 3II).

### Complex C1 Induced the Activity of Caspases 7 and 9

**Fig 4:**
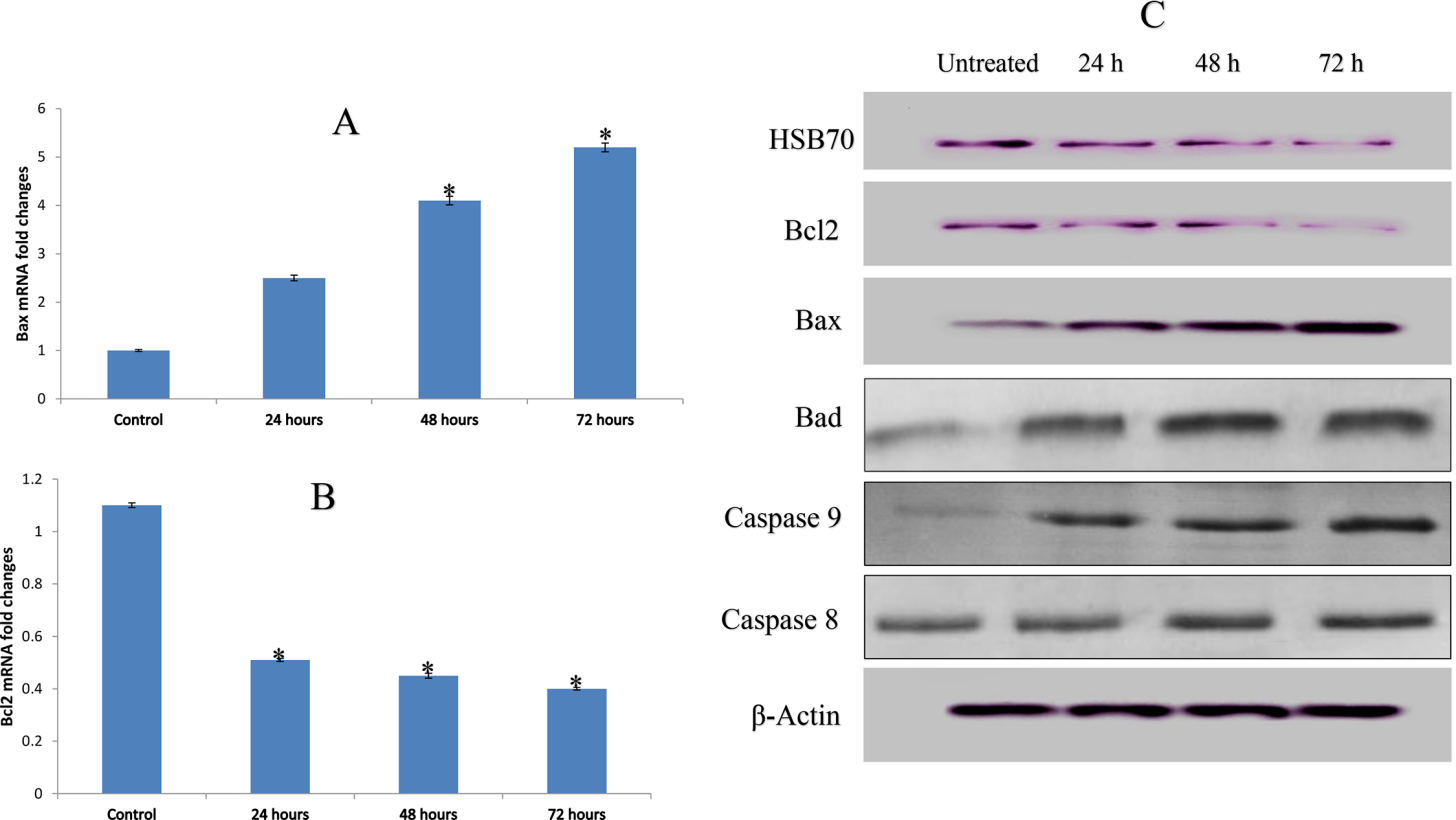
**(A, B) Quantitative study of gene expression, (C) western blot analysis of protein markers of the apoptosis pathway in MCF-7 treated cells with a concentration of 2.5 μg/mL (IC**_**50**_**) respectively**. The expression of Bax **(A)** and Bcl2 **(B)** mRNA in MCF-7 cells treated with a concentration of 2.5 μg/mL (IC_50_) of compound C1 after 24, 48, and 72 hours. The results revealed a significant elevation and a decrease in the expression of the Bax and Bcl2 genes, respectively. **(C)** showed that compound C1 increased protein expression levels of Bax, Bad and decreased the Bcl2, HSP70. Values are expressed as the mean ± the standard deviation, n = 3 per treatment group. Statistical significance was equal to *p < 0.05.

A complex of cascades which include different proteinase caspases are fundamental part of energy-consuming development of apoptosis. The activities of caspases 7, 8 and 9 are important biochemical characteristics of apoptotic signaling [42]. The abovementioned results showed some morphological and biochemical features of apoptosis in compound C1-treated cells. Therefore, in this study, the activities of these caspases were measured to confirm the apoptotic effect of compound C1. The results indicated that all caspase activity increased in MCF-7 cells in a time-dependent manner as a result of compound C1. Fig 1II illustrates that caspase 7 activity increased after 48 and 72 hours of treatment, while caspase 9 activity increased significantly after 24, 48 and 72 hours treatment (*p* < 0.05). Caspase 8 activity also increased in the treated cells compared with the control cells, although this increase was not statistically significant (Fig 1II). To further determine whether apoptotic induction effect of compound C1 was mediated by caspases we measured the protein expression level of two initiator caspases (caspase 8 and 9). As shown in Fig 4C caspase 9, a main regulator of mitochondrial apoptosis pathway, was up-regulated in time dependent mode, while the expression level of caspase 8 remained unchanged.

### Compound C1 Induced Expression of Protein markers of Apoptosis at the Gene and Protein Expression Level

Alterations in expression of apoptosis-related markers has important role in the control of mitochondrial pathway of apoptosis [39]. The changes in MMP and cytochrome *c* release in treated MCF-7 cells led us to further establish the role of mitochondria in C1-induced apoptosis at the protein level by western blot. Among the proteins that are linked to apoptosis pathways, the protein expression levels of anti-apoptotic proteins (e.g., Bcl2) and pro-apoptotic proteins (e.g., Bax, Bad, and HSP70) were analyzed (Fig 4C). The expression of Bax and Bad was up-regulated by compound C1, while the expression of Bcl2 and HSP70 proteins was significantly suppressed.

Real time PCR analysis was used to assess the mRNA expression levels of Bax and Bcl2 (apoptosis marker genes). The results of the analysis revealed that Bax expression levels were low in untreated MCF-7 cells and that they increased significantly following 24, 48, and 72 hours of treatment (*p* < 0.05) (Fig 4A). The activity of the anti-apoptotic protein Bcl2 was significantly decreased after 24, 48, and 72 hours of treatment (*p* < 0.05) (Fig 4B).

### Breast CSCs Were Identified Based on CD Markers

**Fig 5:**
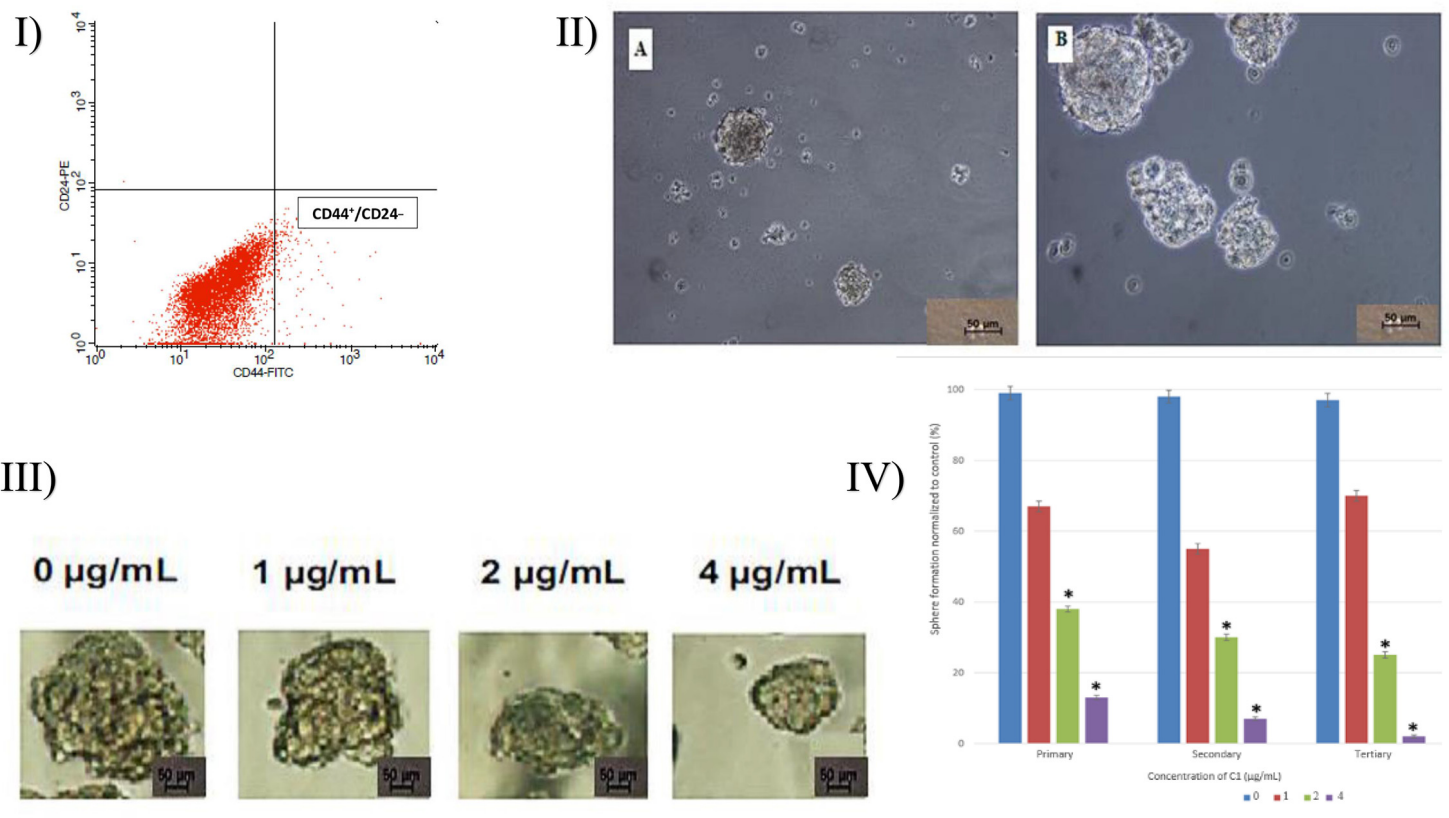
**I) Identification of MCF-7 cancer stem cells, II) Mammosphere formation of MCF-7 cancer stem cells, III) reduction in the size of primary mammospheres in treated MCF-7 cancer stem cells, IV) inhibition effect of compound C1 on primary, secondary, and tertiary mammosphere-forming units. I:** The expression of CD44+ and low levels of CD24 during a quadrant examination. (CD44^+^/CD24^-/low^) were used to identify MCF-7 CSCs. **II: (A)** and **(B)** show the size of mammospheres containing MCF-7 cancer stem cells on day 5 and day 7, respectively. **III:** Complex C1 reduced the size of the primary mammospheres in treated MCF-7 cancer stem cells. **IV:** The second and third passages derived from Complex C1-treated, primary mammospheres yielded smaller numbers of spheres compared with the control. The size of the mammospheres was estimated using V = (4/3)πR3. Complex C1 inhibited mammosphere formation and prevented the self-renewal of MCF-7 in (1) primary, (2) secondary, and (3) tertiary mammosphere-forming units. Data are shown as the mean ± the standard deviation (n = 3). Statistical significance is expressed as *p < 0.05.

A growing body of evidence have been recently supported the assumption that breast cancer cells with high or low expression of specific surface markers are capable to form tumor. In breast cancer, CD44+/CD24_/low cells, posess stem cell–like properties [43]. Isolation of breast CSCs was performed by cell sorting according to the expression of the cell surface markers CD44 and CD24 (i.e., CD44+/CD24-/low) (Fig 5I). These cells were then grown to form mammospheres. MCF-7 BCSCs were identified based on the expression of CD44 and low expression of CD24.

### Complex C1 Inhibited the Growth of Mammospheres

Mammosphere formation, which is a property of breast progenitor cells, was observed under a Nikon Eclipse TE2000-S microscope, and images were obtained with MetaMorph 7.6.0.0 (Fig 5IIA and 5B). It has been reported that mammary stem/progenitor cells are able to grow in serum-free culture medium and form non-adherent spherical clusters of cells, known as mammospheres. The MCF-7 CSC mammospheres were exposed to different concentrations of complex C1 to assess the suppressive effects of agent C1 on the *in vitro* formation of spheres. As shown in Fig 5III, complex C1 inhibited the growth of spherical clusters of BCSCs, and the number of spheres decreased significantly with increasing concentrations of compound C1 (*P* < 0.01) (Fig 5IV). According to these findings, C1-treated cells were not able to differentiate into multiple lineages and were not able to produce subsequent spheres.

### Complex C1 Reduced the ADH-positive Cell Population

**Fig 6:**
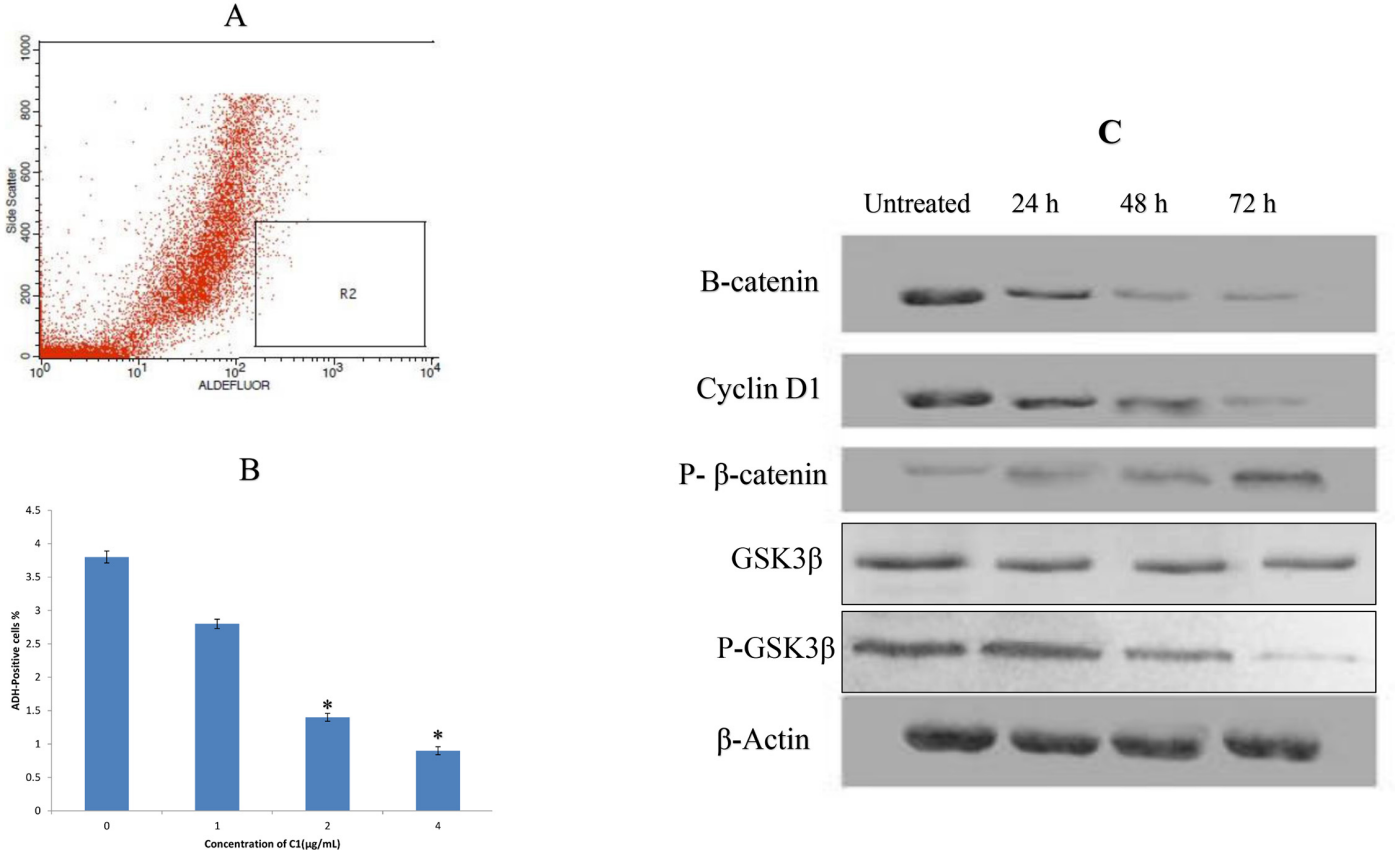
**(A, B) Aldefluor assay of MCF-7 cancer stem cells, (C) western blot analysis of protein markers of the Wnt/β-catenin self-renewal pathway in MCF-7 treated cells with a concentration of 2.5 μg/mL (IC**_**50**_**).** An aldefluor assay buffer composed of an ADH substrate and BODIPY-aminoacetaldehyde (1 μmol/Lper 1×106 cells) was used to incubate single cells from cell cultures at 37°C for 50 minutes **(A)** a cell population (R2) with high ADH activity contained more Mammary stem/progenitor cells **(B)** a quantitative analysis of the inhibitory effect of C1 on ADH-positive cell populations MCF-7. Here, complex C1 at concentrations of 1, 2, and 4 μg/mL were used to treat cancer stem cells for 4 days, which were later subjected to Aldefluor assay and flow cytometric analysis. Complex C1 decreased the percentage of ADH-positive cells. Compound C1 decreased protein expression levels of β-catenin, cyclin D1, and PGSKβ and increased the phospho-β-catenin Ser33/Ser37/Thr4. Data are expressed as the mean ± the standard deviation. (n = 3). Statistical significance wasequal to *p < 0.05.

A cell population with high ADH enzymatic activity may be used to detect mammary stem/progenitor cells *in vitro*. ADH activity stimulates self-renewal of MCF-7-derived CSCs, and therefore, ADH activity was assessed using the aldefluor assay (Fig 6A). Treatment with different concentrations of compound C1 (1, 2, and 4 μg/mL) significantly reduced the ADH-positive population of MCF-7 CSCs compared with the control. This result further supports the concept that compound C1 reduced the population of breast MCF-7 CSCs. Furthermore, the preferred activity of compound C1 in regards to MCF-7 CSCs was confirmed, as this compound was unable to affect C1- treated MCF-10A cells [38], while it inhibited MCF-7 CSCs effectively at concentrations of 1, 2, and 4 μg/mL (Fig 6B).

### Compound C1 induced Expression of Protein markers of the Wnt/β-catenin Self-renewal Pathway

Glycogen synthase kinase‐3 β (GSK3-β) mediates Wnt signalin pathway. Phosphorilated GSK3-β results in translocation of nonphosphorylated β-catenin from cytosol to nucleus and subsequent Wnt signaling cascade formation as a stem cell self-renewal pathway [44]. Based on our findings, the expression levels of β-catenin, p-GSK3β, and cyclin D1 were down-regulated in a time-dependent manner, but the expression level of p-β-catenin (Ser33/Ser37/Thr41) was up-regulated. The expression level of GSK3-β in treated MCF-7 cells remained almost unchanged. (Fig 6C).

## Discussion

Apoptosis, which is a controlled cellular pathway, regulates the homeostasis between cell proliferation and cell death. The reduction in the occurrence of apoptosis disrupts this balance, causes cancer and promotes its progression [45]. Therefore, the study of the key regulators of apoptosis will lead to the introduction of effective therapeutic agents with high efficacy and fewer undesirable side effects [46, 47]. This process can be initiated by a number of stimuli, such as radiation, drugs, deficiency of hormones or growth factors, and toxins [48].

In the present study, we discussed a few recognized components of the molecular mechanisms of apoptosis such as the Bcl-2 family, the release of cytochrome *c*, cycle interfaces, caspase regulation, changes in the mitochondrial membrane potential, and annexin V staining of phosphatidylserine residues on the outer cell membrane. Annexin V staining was conducted in order to quantify the number of apoptotic cells. In the presence of calcium, annexin V forms strong and selective bonds with the phosphatidylserine found in the external membranes of cells [49]. Externalized PS residues can be detected using fluorescein isothiocyanate (FITC), an active fluorescence dye, and conjugated annexin V. Externalized PS residues imply the loss of plasma membrane asymmetry in apoptotic cells [50]. In this experiment, the emission of green fluorescence distinctly illustrated that C1 promoted apoptosis in MCF-7 cells. The cell cycle is the process used by cells to divide and replicate themselves [51]. Under ordinary circumstances, any errors in the cell cycle are either corrected, or the cell is destroyed via apoptosis. However, in cancer cells, this process malfunctions, which causes uncontrolled cell proliferation [52]. Consequently, the introduction of drugs that target cell cycle regulation would cause cells to arrest within various stages of the cell cycle and lead to chemoprevention and advancements in treatments for cancer [53].

The results of this study indicate that the cytotoxicity induced by C1 in MCF-7 cells might be the result of its ability to initiate cell cycle arrest in the G0/G1 phase via overexpression of p21 and p27 proteins. The cyclin-dependent kinase activity inhibitors, p21 and p27 proteins, are known to have significant role in cell division [54]. Therefore, the agent with the capability to induce apoptosis and upregulate p21 and p27 proteins can be considered as promising anticancer agents. Several studies have suggested that caspases, which are specific proteases, play an important role in the induction and implementation of apoptosis [55]. Apoptosis is initiated by either a death receptor-mediated extrinsic pathway or a mitochondrial-mediated intrinsic pathway. The death receptor-mediated extrinsic pathway is triggered by transmembrane receptor-mediated interactions including those of Fas, TNF, and TRAIL. In contrast, the mitochondrial-mediated intrinsic pathway is initiated by intracellular signals that eventually cause the mitochondrial membrane potential (MMP) to become disrupted, which leads to the release of cytochrome *c*, Smac and other mitochondrial proteins [56, 57]. The formation of the apoptosome, which is a caspase-activating complex, is activated by Cytochrome *c*, which stimulates the production of caspase 9. This leads to the eventual activation of the remaining caspases (e.g., caspases 3 and 7) that are a part of the apoptotic cascade, and ultimately, apoptosis occurs.

The tumor necrosis factor (TNF) family of death receptor-mediated apoptosis pathways is connected to caspase 8 even though the activation of caspase 8 is seen both upstream and downstream of the mitochondria [58, 59]. In this study, the significant increase in the activity of caspases 7 and 9 indicated the caspase-dependent apoptotic effect of C1 in MCF-7 cells. Additionally, a time dependent increase in caspases 9 expression at protein level imply that apoptosis was initiated by the intrinsic pathway in our study.

The mitochondrion is a possible source of reactive oxygen species (ROS), which allows this organelle to play an important role in the production of ROS [57, 60]. Changes in the mitochondrial membrane potential and subsequent cytochrome *c* release are disrupted by ROS that oxidize the pores of the mitochondria [61]. In this study, the results of a high content screening analysis revealed that C1 affected mitochondria, instigated the loss of MMP, and increased the release of cytochrome *c*. This increase may have been caused by mitochondrial membrane transition pores (MPTP). Bax is a member of the Bc12 family of proteins. When Bax moves into the mitochondria, it exerts devastating effects [62]. The Bcl2 family of proteins controls the mitochondrial-mediated apoptosis pathways [63]. For example, Bcl2 prevents the incidence of apoptosis by preventing the release of cytochrome *c* from mitochondria [53]. This means that the ratio of Bax to Bc12 is critical for the regulation of apoptosis [64]. The connection between the Bax/Bcl2 ratio and the apoptotic effects of C1 was illustrated in the treated MCF-7 cells. HSP70 is another crucial protein that plays a role in apoptosis. In this study, the expression of HSP70 decreased in a time-dependent manner. Additionally, the relationship between Bax and Bcl2 was demonstrated at the gene level in this *in vitro* study. Bad, BH3-only proteins, is the other cytosolic proapoptotic member of of Bc12 family of proteins. Dephosphorylation of Bad by the Ca21-sensitive phosphatase calcineurine or the protein phosphatase 1a (PP1a) cause translocation of Bad from cytosole to mitochondria and subsequent binding with Bcl-xL, release of cytochrome c and occurrence of apoptosis [65]. In this study, the expression of Bad in treated MCF-7 cells increased in a time-dependent manner.

As cancer biology is studied in order to understand the mechanisms that cause tumorigenesis and progression, another theory was suggested that tumors must contain some cells with more permanence and that these cells underlie resistance to chemotherapy and cancer recurrence. This definite subpopulation of cells is known to consist of cancer stem cells (CSCs), which sustain tumor growth. CSCs were initially defined in people with acute myeloid leukemia (AML) [66]. CSCs have now been recognized in a variety of malignant tumors, such as breast [24, 67]_,_ lung, gastric, prostate, colon, pancreatic, liver, and brain cancers; melanoma; multiple myeloma; and medulloblastoma [20]. However, the accurate identification of CSCs has continued to be investigated. Many have mixed views on this topic. Rossi et al suggested that mutations in genes that regulate processes that govern proliferation vs. apoptosis, self-renewal, and differentiation of progenitor or stem cells may give rise to a loss of immunogenicity, avoidance of apoptosis and the subsequent generation of CSCs [20,68]. According to Visvader and Lindeman, the atypical reactivation of pathways that involve Wnt, Notch and Hedgehog in progenitor or stem cells or the malignant transformation of these cells may lead to the generation of CSCs [69]. Cancer stem cells were further identified after the formation of tumors in immunodeficient animal models, where tumors with the same phenotypic characteristics of the principal tumor were observed after the transplantation of cells [70–74]. The successful isolation of BCSCs is the first step *in vitro* to direct BCSCs in therapeutic approaches [23]. Methods such as mammosphere culture, cell marker-based isolation and ADH tests have been used for the isolation and identification of BCSCs *in vitro*. The culture of MCF-7 cells in serum- free medium resulted in the failure of differentiated cells to survive, whereas breast cancer stem/progenitor cells were able to expand and form mammospheres [75–77]. The proliferaion of CSC is to the first step in studding of CSC. Growgh of CSC-enriched population cells in the serum-free suspension cultures is the best way to profilate them. Breast cancer stem cells grow as spherical colonies in this condition.Therefore, studies of mommospheres reflect the propeeties of BCSCs [75].

Cell surface markers such as CD44^+^/CD24^-/low^ have been used to separate mammary stem/progenitor cells from differentiated breast cancer cells [78]. Synthetic compounds such as fenretinide and the dopamine antagonist thioridazine were reported to selectively prevent the generation of colonies of CD34+ AML cells and LSCs, respectively. Fenretinide and the dopamine antagonist thioridazine have no effect on normal CD34+ cells and normal hematopoietic stem cells, respectively [79–81]. Our results show that C1 significantly inhibits the formation of mammospheres. Therefore, this finding suggests that C1 might eradicate MCF-7-derived breast cancer stem cells and also confirms the results of other studies that found that mammospheres are composed principally of cancer stem cells [82]. In order to further confirm the presence of BCSC populations within breast cancer, the MCF-7 cell line was used in an aldefluor assay to offer a more definitive demonstration for the targeting of BCSCs by C1. It is agreed that ADH expression can be used as an indicator to isolate subpopulations of cells that exhibit stem cell properties from cancer cell lines [77]. ADH is a detoxifying enzyme that is associated with stem cell differentiation and stem cell self-protection via its involvement in intracellular retinoic acid generation [83, 84]. Based on our results, complex C1 could selectively suppress the ADH-positive cancer cells *in vitro*. Strategies in BCSC-targeted treatment involve either the pursuit of the self-renewal signaling pathway of BCSCs or the direct killing of BCSCs [85, 86]. Our results showed that monobenzyltin complex C1 has the ability to down-regulate the Wnt/β-catenin self-renewal pathway via a reduction in β-catenin and cyclin D1 and an increase in β-catenin Ser33/Ser37/Thr4 in treated cells. Additionally, the degradation of the proteasome through activity of GSK3β is the possible mechanism of compound C1 to target MCF- derived CSCs.

## Conclusion

Compound C1 selectively promotes the death of tumor cells through apoptosis and cell cycle arrest. In this study, we investigated the molecular processes that contribute to the C1-induced apoptosis of MCF-7 cells. The C1-induction of G1 cell cycle arrest was suggested to be through upregulation of p21 and p27 proteins. Our results revealed that C1 controls the MMP via the up regulation of Bax and Bad, and the down regulation of Bcl2 in addition to the control of cytochrome c release from the mitochondria to the cytosol, which then triggers caspase 9. Once activated, caspase 9 promotes the activity of caspase 7, which cleaves specific substrates that are responsible for the initiation of apoptosis. This study also verified that phosphatidylserine bound to annexin V translocates from the outer surface of the plasma membrane. Moreover, C1 exerts targeted effects on MCF-7-derived CSCs, as determined by the mammosphere formation assay and marker-based assays (e.g., CD44^+^/CD24^-/low^ and ADH). Furthermore, the C1-induced down-regulation of the Wnt/β-catenin self-renewal pathway was introduced as a potential mechanism of action of this compound. This study supports the chemo preventive characteristics of complex C1 for breast cancer with a strong basis for clinical assessment in the future.
